# An introductory laboratory class in sonochemistry

**DOI:** 10.1016/j.ultsonch.2023.106691

**Published:** 2023-11-07

**Authors:** Grzegorz Matyszczak

**Affiliations:** Warsaw University of Technology, Faculty of Chemistry, Noakowski Street 3, 00-664 Warsaw, Poland

**Keywords:** Sonochemistry, Sonocatalysis, Education, Acoustic cavitation, Calorimetry, Sonochemical efficiency

## Abstract

This article proposes a substantive scope and scenario of a laboratory class that introduces students to the field of sonochemistry. The class requires only basic laboratory equipment - typical laboratory glassware like graduated pipettes and conical flasks, as well as simple inorganic chemicals. It is designed to acquaint students with fundamental aspects of sonochemistry. In the qualitative aspect, they will conduct and observe some sonochemical reactions like a synthesis of hydrogen peroxide and ultrasound-assisted degradation of toxic chromates(VI) which will demonstrate the indirect consequences of water sonolysis which is the most basic sonochemical reaction, as well as they will illustrate the applications of sonochemistry. In the quantitative aspect, students will learn about how to measure the power of ultrasound and the sonochemical efficiency of the reaction and will conduct experiments allowing for the calculation of these parameters. Finally, an introduction to and demonstration of the sonocatalytic effect is planned. An evaluation system, consisting of a report and test, is also proposed.

## Highlights

• Scope and scenario of introductory laboratory class in sonochemistry is presented.
• Class requires basic lab equipment: scale, spectrophotometer, magnetic stirrer.
• Mechanical and chemical consequences of acoustic cavitation are demonstrated.
• Students learn how to determine ultrasound power and sonochemical efficiency.
• Students learn how to conduct sonocatalytic degradation of toxic CrO_4_^2-^.

## 1 Introduction

In most general words, the scope of sonochemistry is the investigation of chemical reactions (i.e. changing the chemical state of matter – breaking and creating chemical bonds) performed in a medium subjected to ultrasounds. Liquid medium contains chemical reactants that undergo chemical reactions induced by ultrasounds. Such medium most commonly is water and the basic reaction that occurs in it due to the action of ultrasound is the water sonolysis.

H_2_O → H**·** + **·**OH

As it is taught in the general and inorganic chemistry courses, the water molecule isn't so easy to break and requires additional energy to destroy it, e.g. by introducing external electric current and performing electrolysis. In sonochemistry, the additional energy comes from ultrasound. The passing of ultrasonic waves through a liquid medium causes fluctuations in pressure inside it which potentially could affect the solubility of gases dissolved in the medium (in the case of reaching negative pressure) and start the nucleation of bubbles. However, the tensile strength in liquid medium is great enough to typically disallow such a phenomenon [1]. Instead, the formation of acoustic bubbles is allowed by discontinuities in liquid medium (e.g. micro-gaps filled with gas or micro-bubbles of distinct origin) [1]. Once formed, the acoustic bubble grows and then, after reaching its resonant size, collapses generating extreme conditions [1]. In the collapsing bubble the temperature rises to thousands of Kelvins and pressure reaches thousands of standard atmospheres [2], [3]. Under these conditions the acoustic bubbles are filled with vapor water and water molecules may be broken.

The history of sonochemistry has almost one hundred years and started with the discoveries of Prof. Wood and Mr. Loomis reported officially in 1927 [3], [4], [5]. However, a rapid development of sonochemistry was seen in the last few decades of exciting investigations. The application of ultrasound in chemistry is as wide as chemistry itself and comprises - among others - organic, inorganic, analytical, polymer, food, and environmental chemistry. Examples for each division are summarized in Table 1.

**Table 1:** Summary of examples of applications of ultrasound across selected divisions of chemistry.

**Chemistry division**	**Examples of applications**	**References**
Organic	Performing organic syntheses in milder than typical conditions, as well as faster than typically.	[6], [7], [8], [9]
Inorganic	Production of nanoparticles of inorganic semiconductors under room conditions.	[2], [3], [10], [11]
Analytical	Sample preparations and pretreatments. Electroanalytical chemistry (for example, allowing improvement in stripping voltammetry).	[12], [13], [14]
Polymer	Ultrasound may affect the properties, such as molecular weight, of synthesized polymers and increase the rates of early-stage reactions.	[15]
Food	In food processing, ultrasound allows the decrease of processing and extraction time, the amount of solvents and energy used, unit operations, and CO_2_ emissions.	[16], [17], [18]
Environmental	Degradation of compounds present in wastewater, such as antibiotics, pesticides, dyes, etc. that are dangerous to the environment and human health.	[19], [20], [21]

As sonochemistry is a very important and rapidly developing field, there is an obvious need to introduce it to the course of study for undergraduate students in disciplines such as chemistry, chemical technology, and chemical engineering. This article presents the laboratory class introducing students to the basic concepts of sonochemistry and allowing them to learn how to perform simple sonochemical experiments. In the form presented in this article, the classes were conducted for students of the Faculty of Chemistry at Warsaw University of Technology as a part of “Laboratory of Chemical Technology” classes in the field of study “Chemical technology” in the sixth semester. The class is designed to take two meetings for c.a. 5 h each, but it can be limited to one meeting as well it can be adapted as a demonstration of issues explained in a standalone, strictly theoretical, lecture.

## 2 Pre-requirements

### 2.1 1. Student's knowledge and skills

The classes were taught to students in the sixth semester, which is rather high in the course of study, however, to perform the designed tasks student needs to have only basic laboratory skills such as weighing and pipetting. All experiments are conducted at room conditions and don’t involve any highly hazardous reagents.

To understand the theoretical introduction to the classes and analyze the results of experiments, student needs to have basic knowledge of general chemistry. She/he should:

- know what is hydrogen peroxide,
- know how pressure affects the solubility of gases in liquids,
- know what are radicals,
- know the Lambert’s law and be familiar with spectrophotometry,
- have general knowledge of chemical kinetics.

The laboratory class presented here could be as well taught at an early stage in the course of study.

## 3 Laboratory equipment

**Fig. 1:**
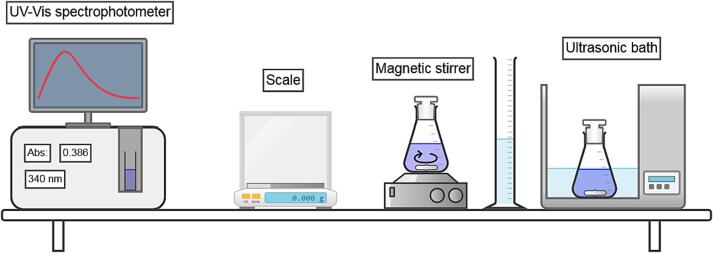
The scheme of required laboratory equipment in the basic form of classes.

To conduct classes in the proposed form the laboratory should be equipped with UV–Vis or Vis spectrophotometer (Fig. 1). The source of ultrasound is an ultrasonic bath. The laboratory centrifuge and magnetic stirrers are also desired, however, they may be omitted because, in the first case, the synthesized powders may be separated by simple filtration and, in the second case, the mixing of reagents may be done with glass baguette.

When it comes to laboratory glass, the classes make use of conical flasks with stoppers, graduated cylinders, pipettes, falcons (centrifuge tubes), quartz cuvettes (for spectrophotometry), magnetic stirring elements, and clamps.

## 4 Course content

### 4.1 The mechanical effect of ultrasonic cavitation

At the beginning of the class, students have an occasion to see the effect of the mechanical action of acoustic bubbles. Before beginning the calorimetric measurements they place a piece of aluminum foil into the water inside the ultrasonic bath. Firstly, the piece of foil is shown to them to demonstrate how it looks before sonication. Students are asked to make a bet on what will happen to the foil after 30 min of sonication. Typical answers say that nothing will happen or that it will disintegrate in some way, or that it will turn white or mat (in general: change its color) due to oxidation on the surface.

**Fig. 2:**
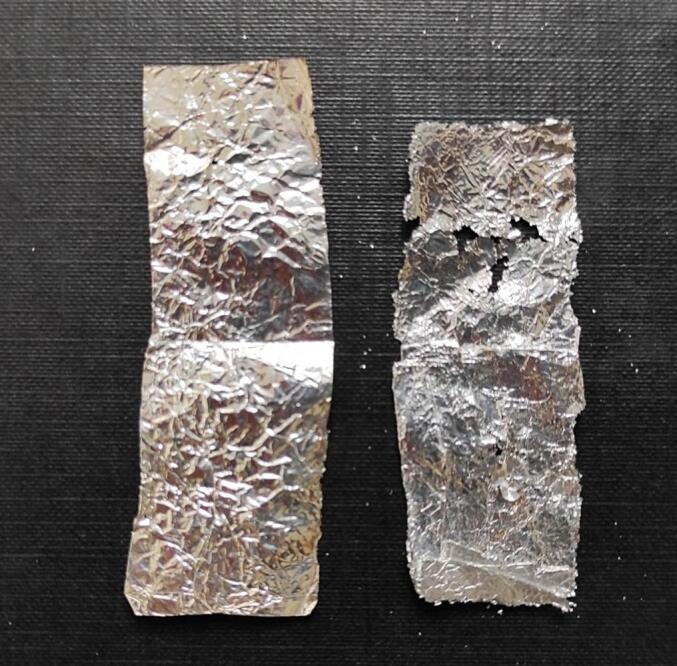
The photograph of aluminum foil (left) before sonication and (right) after 30 min of sonication.

During the calorimetric measurements, the foil undergoes mechanical destruction and it is indeed disintegrated. In the water that fills the ultrasonic bath small pieces of aluminum may be seen and the foil has holes (Fig. 2).

Through this experiment, students learn about the mechanical consequences of the collapse of an acoustic bubble. It may cause the formation of shock waves or, in this case, microjets. The latter are formed when the bubble meets a flat solid surface (in this case the aluminum foil) of size greater than the resonant size of the bubble. Under such circumstances, the collapse of a bubble is nonspherical. The formed micro jets reach velocities of up to hundreds of meters per second and hit the surface of the foil causing her erosion.

### 4.2 Calorimetric determination of ultrasound power

**Fig. 3:**
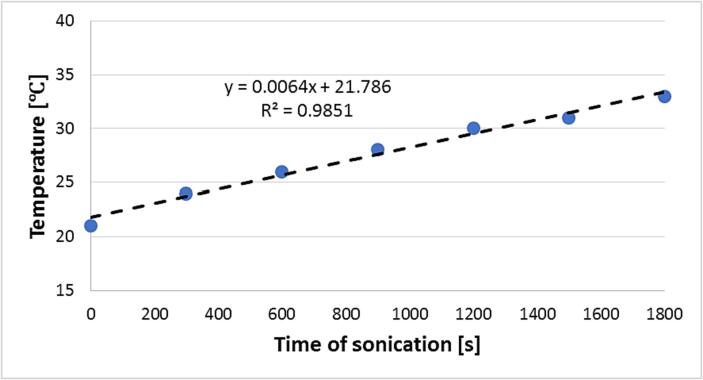
Sample plot of experimental data obtained in measurements of the temperature of water inside ultrasonic bath under sonication.

In parallel with the previous task, students conduct measurements allowing for the determination of ultrasound power generated by the ultrasonic bath. They measure c.a. 1 dm^3^ of tap water and place it in the ultrasonic bath. After turning the ultrasound ON they note the temperature of the water inside the bath every 5 min for, say, 30 min. The ultrasonic bath may be equipped with a temperature sensor or the measure of temperature must be done externally. An example of experimental points that may be obtained is presented in Fig. 3.

The points are organized in linear trend so students have to fit them with linear function. Then they may calculate the power of ultrasound generated by an ultrasonic bath according to the following equation:

(1) $$P=C_{p}M\frac{\mathrm{dT}}{\mathrm{dt}}$$

where: P – the power of ultrasound [W], M – the mass of liquid in the bath [g], T – temperature [°C or K], t – time of sonication [s], C_p_ – specific heat of used liquid [J∙K^−1^∙g^−1^].

The C_p_ is taken from literature data, M is known (c.a. 1 kg) but the student's task is to determine the value of dT/dt from obtained experimental points. They must recognize that dT/dt is the slope of the fitted linear function. After that, they can calculate the value of ultrasound power and additionally determine the efficiency η of ultrasonic bath from equation no. 2:

(2) $$\eta =\frac{P}{P_{\mathrm{nom}}}∙100\%$$

where: η – efficiency of ultrasound source [%], P – power of ultrasound [W], P_nom_ – nominal power of ultrasound source [W].

Students are instructed that all of the energy of generated ultrasound is converted to heat in the ideal case. They are also informed that a portion of the ultrasonic wave dissipates to the surroundings thus the power of ultrasound is less than 100 %. Taking into account that the nominal power of the ultrasonic bath used in performed classes was 60 W and typically the measured power of ultrasound was of value c.a. 28 W, the efficiency of the ultrasonic bath determined by students is slightly below 50 %. They are expected to conclude that the estimated values of ultrasonic power and efficiency are biased due to the lack of thermal isolation and to the dissipation of heat to the surroundings.

### 4.3 Determination of sonochemical efficiency of hydrogen peroxide generation

The next task for students is to perform a sonochemical synthesis of hydrogen peroxide, through the recombination of hydroxyl radicals generated in water sonolysis:

2 **·**OH → H_2_O_2_.

**Fig. 4:**
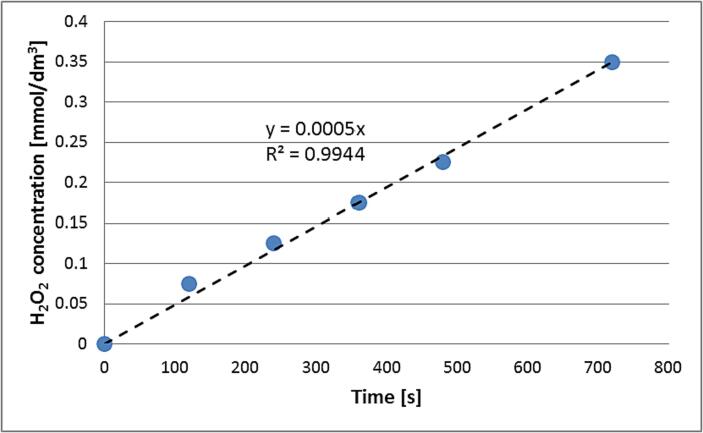
A typical plot of measured concentrations of hydrogen peroxide during sonochemical synthesis.

Students measure a portion of distilled water (e.g. 20 cm^3^) and place it in a conical flask equipped with a stopper. Then they place the flask in an empty ultrasonic bath and add tap water to the bath so the levels of liquids in the bath and flask are c.a. the same. The flask may be additionally protected with a clamp in case it falls over. Then they start the sonication and measure the absorbance of water in the flask every 2 min for several minutes (14–18 min is typically enough) at 240 nm using a spectrophotometer (method 1). The quartz cuvette must be used because 240 nm lies in the UV region. The extinction coefficient of H_2_O_2_ at 240 nm is 40 dm^3^·mol^−1^·cm^−1^. Students measure the absorbance during the reaction and at the moment of measurement, they turn the sonication OFF to stop the process. After each measurement, the taken portion of distilled water is returned to the flask in the bath and the ultrasound is turned ON for another 2 min. After applying Lambert's-Beer's law students may determine the concentrations of hydrogen peroxide during sonochemical synthesis (Fig. 4).

The H_2_O_2_ concentration may be also determined using the indirect iodide method (method 2). The procedure is similar as in the case of direct method 1, however, an excess of potassium iodide must be dissolved in water that is then subjected to ultrasound. The absorbance is measured at wavelength c.a. 350 nm. The iodide anions react with generated H_2_O_2_ and form molecular iodine I_2_ that further is transformed to I_3_^-^ anions responsible for the yellow color of the mixture (color may be hardly observable due to dilution but should be detectable by spectrophotometer):

H_2_O_2_ + 2 I^-^ → I_2_ + 2 OH^–^.

I_2_ + I^-^ → I_3_^-^.

The sonochemical generation of hydrogen peroxide is assumed to follow zeroth order kinetics [22], [23], [24], so the students have to fit linear function y = a·x to the experimental points:

(3) $$\frac{d[H_{2}O_{2}]}{\mathrm{dt}}=k$$

(4) $$\left[H_{2}O_{2}\right]=kt+{\left[H_{2}O_{2}\right]}_{0}=kt$$

where: [H_2_O_2_] – concentration of hydrogen peroxide [mol∙dm^−3^], k – kinetic constant for zeroth order reaction [mol∙s^−1^∙dm^−3^], t – time [s], [H_2_O_2_]_0_ – initial concentration of hydrogen peroxide [mol∙dm^−3^].

The slope is the rate of reaction of generation of H_2_O_2_ and its value is used in the calculation of sonochemical efficiency (SE) of this reaction, according to equation no. 5:

(5) $$\mathrm{SE}=\frac{Y∙V}{P}$$

where: Y – yield of reaction [mol∙dm^−3^ or mol∙s^−1^∙dm^−3^ – for periodic or continuous process respectively], V – volume of reaction mixture [dm^3^], P – ultrasound power [W].

In this case, the yield of reaction Y is the rate of reaction of H_2_O_2_ generation. The reaction mixture is known from the start. The value of ultrasound power is determined in the previous point. The sonochemical efficiency calculated for the continuous process (as it is in this case) has unit mol∙s^−1^∙W^−1^ or mol·J^−1^ and will tell the students how much product (in moles) may be obtained by 1 J of ultrasonic energy.

### 4.4 Sonochemical production of inorganic semiconductor

At this point, students conduct another sonochemical reaction. It will be a synthesis of inorganic compound with semiconducting properties, such as SnS, SnS_2_, ZnS, Bi_2_S_3_, etc. The reaction may be performed in distilled water starting from a salt of corresponding metal (e.g. Bi(NO_3_)_3_) and thioacetamide. It will decompose to, among others, H_2_S which will react with metal cations forming insoluble sulfide:

CH_3_CSNH_2_ + 2 H_2_O → CH_3_COOH + NH_3_ + H_2_S.

Sn^4+^ + 2 H_2_S → SnS_2_ + 4H^+^.

**Fig. 5:**
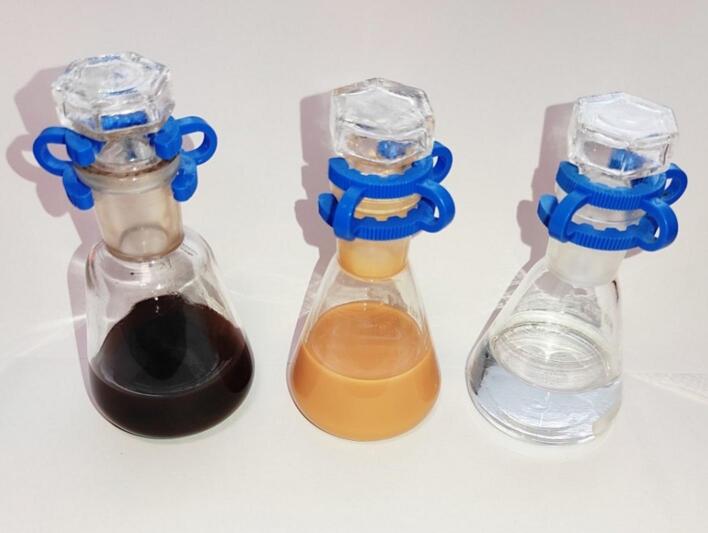
Photograph of conical flasks with reaction mixtures (water, metal salt, thioacetamide) from left to right: after 100 min of sonication in the synthesis of Bi_2_S_3_, after 100 min of sonication in the synthesis of SnS_2_, and before sonication.

The students have to weigh the reagents in amounts corresponding to 2 mmol of metal cation (e.g. Sn^4+^) and 5 mmol of thioacetamide – however, the amounts may be varied if possible. Reagents are dissolved in 20 cm^3^ of distilled water and placed in a conical flask. Then the flask is placed in the empty ultrasonic bath and the bath is filled with tap water so the levels of liquids in the flask and bath are approximately the same (like in the case of H_2_O_2_ synthesis). The flask may be additionally protected with a clamp. Then the sonication is started and it should last for several dozen minutes to observe the precipitation of powder. This moment may vary for different compounds and, for example, in the case of SnS_2_ synthesis the yellow color is seen after c.a. 30–40 min of sonication (Fig. 5).

After the end of the reaction, the product may be purified by several centrifugations or by simple filtration. Students may also leave the purified powder to dry and weigh it to determine the sonochemical efficiency of the synthesis of the inorganic compound. In this case, the process is periodic so the sonochemical efficiency may be calculated according to equation no. 6:

(6) $$\mathrm{SE}=\frac{m_{\mathrm{dry}}}{P}$$

where: m_dry_ – a mass of dried purified product of synthesis [g], P – ultrasound power [W].

Students may also compare the obtained sonochemical efficiency with the one for the synthesis of H_2_O_2_ and make conclusions. The efficiency of synthesis of inorganic semiconductor at first glance should be lesser than in the case of H_2_O_2_ generation because H_2_O_2_ is created in fast recombination of slowly produced hydroxyl radicals **·**OH. However, such a comparison isn’t so straightforward. The generation of H_2_O_2_ is due to the recombination of hydroxyl radicals produced in water sonolysis but the formation of inorganic sulfide is due to the reaction of metal cations with hydrogen sulfide produced in the hydrolysis of thioacetamide. The hydrolysis is sped up by the heat generated by acoustic cavitation on the microscale. Taking into account that some radicals may still react with thioacetamide to form the hydrogen sulfide, the sonochemical efficiency for inorganic semiconductor should be greater than the one for H_2_O_2_ generation.

A portion of purified powder of inorganic semiconductor, either dried or in suspension, may be used in the next task.

### 4.5 Sonocatalytic experiment – Degradation of CrO_4_^2-^ anions

**Fig. 6:**
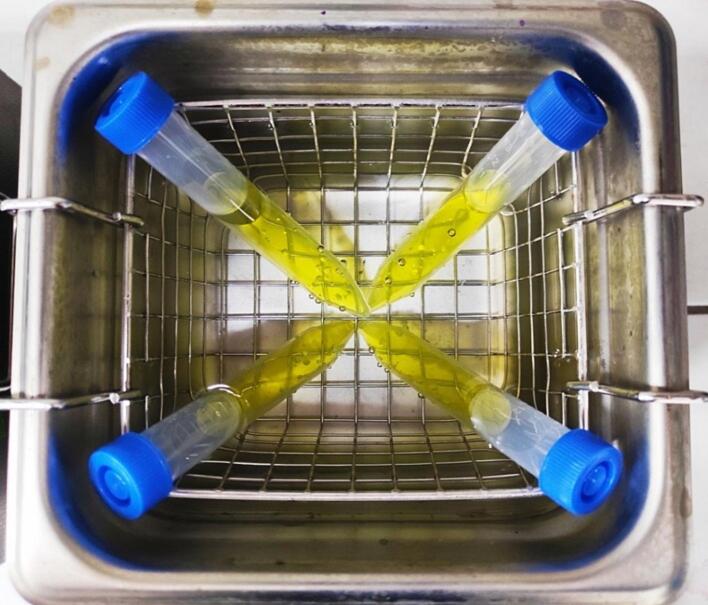
The placement of tubes in an ultrasonic bath in the sonocatalytic degradation of chromates.

In this task, students use sonochemically synthesized inorganic compounds in the sonocatalytic degradation of toxic chromates(VI). They prepare a solution of K_2_CrO_4_ in distilled water with known concentration, which can be controlled by absorbance measurements at 373 nm. Then they add a portion of inorganic compound to the solution and stir it under dark conditions for c. a. 20–30 min to ensure the establishment of absorption–desorption equilibrium which is crucial to let the process work. Then they take four tubes and after that time they pipette a certain volume (e.g. 8 cm^3^) of suspension of sonocatalyst in the solution of chromate to each tube. Four tubes are then closed with stoppers and placed symmetrically in the ultrasonic bath (Fig. 6) and the bath is filled with tap water so the levels of liquids in the bath and tubes are approximately the same. The sonication is turned ON and the ultrasonic bath must be kept under dark conditions from this moment up to the end of the process, which may last e.g. 100 min to let observe the degradation. After that, the sonocatalyst is separated from the solution by centrifugation or filtration. The absorbance measurements at 373 nm (maximum of absorption of K_2_CrO_4_ aqueous solution) before and after sonocatalytic degradation let the students calculate the degree of degradation (equation no. 7):

(7) $$\mathrm{CR}=\frac{A_{O}-A_{E}}{A_{O}}\cdot 100\%$$

where: CR – color removal [%], A_0_ – absorbance before the process, A_E_ – absorbance after the process.

The students should observe that the degradation takes place and that it is strongly dependent on the position of the tube in the ultrasonic bath even though they are placed approximately symmetrically. Thus students learn that ultrasound may be utilized in another chemical application and that the position of the vessel in the bath should be optimized or kept the same among experiments to ensure comparability of results.

This experiment may be performed as an illustration for a theoretical explanation of sonocatalysis. In the lecture or from the laboratory manual students may learn about three cooperating mechanisms of sonocatalysis: thermal excitation of semiconductor by heat emitted during the collapse of acoustic bubbles, photo-excitation of semiconductor by light generated by sonoluminescence, and promotion of acoustic cavitation by semiconductor particles.

## 5 Student evaluation

The students were evaluated based on the results of the test (0 – 5 points) that was conducted in the meantime of laboratory experiments (e.g. during the synthesis of an inorganic compound) and the report (0 – 5 points). The test included two simple open theoretical questions e.g. about what is acoustic cavitation and what reactions take place during the sonochemical generation of H_2_O_2_ (each for 1 point) and one calculation task (for 3 points) consisting in e.g. calculation of the amount of product of sonochemical reaction (in moles or grams) given reaction volume, sonochemical efficiency, and ultrasound power. The report included calculations based on collected experimental data of values of the following quantities: ultrasound power (1 point), the efficiency of the ultrasonic bath (1 point), the sonochemical efficiency of generation of hydrogen peroxide (2 points), and a statement of conclusions (1 point). The examples of test tasks with scoring and the proper solutions are presented in Table 2.

**Table 2:** Test tasks and solutions with scoring.

**Task type**	**Content**	**Desired solution**	**Scoring**
Theoretical question no. 1	Please write the two most important chemical reactions that take place during the sonochemical generation of H_2_O_2_.	Water sonolysis:	0.5 points for each reaction.
		H_2_O → H**·** + **·**OH	Total 1 point.
		Radicals recombination: 2 **·**OH → H_2_O_2_	
Theoretical question no. 2	Please give the formula used in the calorimetric determination of ultrasound power and explain the notations within (include units).	Formula:$P=C_{p}M\frac{\mathrm{dT}}{\mathrm{dt}}$	0.5 points for the correct formula.
		where: P – the power of ultrasound [W], M – the mass of liquid in the bath [g], T – temperature [°C or K], t – time of sonication [s], C_p_ – specific heat of used liquid [J∙K^−1^∙g^−1^].	0.1 point for each correct symbol together with the unit.
			Total 1 point.
Calculation task	Synthesis of ionic liquid (molar mass 300 g/mol) is carried out in a sonochemical reactor of efficiency 60 % and nominal power 50 W. The sonochemical efficiency of this reaction is 1 μmol∙s^−1^∙W^−1^. Calculate the mass of the product and the energy demand (kWh) after one week (seven days) assuming a continuous process.	First, let’s calculate the ultrasound power:	1 point for proper calculation of the
		$P=P_{\mathrm{nom}}∙\eta$	value of ultrasound power.
		P=50W∙60%	1 point for correct calculation of the mass of the product.
		P=30W	1 point for correct calculation of energy consumption.
		Then, let's use it in the calculation of the mass of the product after 7 days of continuous process, utilizing the information about sonochemical efficiency:	Total 3 points.
		$\mathrm{SE}=\frac{Y∙V}{P}$	
		$\mathrm{SE}∙t=\frac{Y∙V∙t}{P}$	
		$\mathrm{SE}∙t=\frac{n}{P}$	
		(n – amount of product [mol])	
		SE∙t∙P=n	
		$\mathrm{SE}∙t∙P∙M_{\mathrm{mol}}=m$	
		$1\frac{\mu mol}{s∙W}∙604800s∙30W$	
		=m	
		m=5443g≅5.4kg	
		Calculation of the energy consumption:	
		E=t∙P	
		E=7∙24h∙50W	
		E=8.4kWh	

The final grade was calculated as the arithmetic average of grades from tests and reports, rounded in ambiguous cases with the benefit for the student (e.g. average 4.75 rounded to 5, not to 4.5). Typically, the distribution of grades was similar to the Gaussian distribution with a mean value near 4.0. The most extreme grades (3.0 and 5.0) were present with c.a. 10 % frequency each. In the case of insufficient grades, the students had a chance to correct the test and/or report.

The laboratory work and reports were done in teams, while the tests were written individually.

## 6 Ending remarks

Herein an introductory laboratory class in sonochemistry is presented, based on the experience of the author. It is intended to demonstrate basic concepts of sonochemistry. Students observe the consequences of the mechanical action of acoustic bubbles (microjetting) and learn how to calorimetrically determine the power of ultrasound based on the heat generated during the collapse of bubbles. They also conduct simple sonochemical reactions, such as generation of hydrogen peroxide and ultrasound-assisted synthesis of powder of inorganic semiconductor. Moreover, they learn how to calculate the sonochemical efficiencies of these reactions. Then they learn how to perform basic sonocatalytic experiment using the previously synthesized semiconductor, on the example of degradation of toxic chromates. The class is planned to take two meetings each c.a. 5 h, but it may be adjusted. Exercises also may be varied and some of them presented in this article may be omitted or modified, according to the needs of the teacher and available laboratory equipment. However, the two most important exercises - calorimetric determination of ultrasound power and measurement of sonochemical efficiency of H_2_O_2_ generation – have low equipment requirements. The proposed scenario and exercises may be further creatively developed. The class may be conducted standalone, as well as it may be linked with theoretical lectures.

## Uncited reference

[5].

## CRediT authorship contribution statement

**Grzegorz Matyszczak:** Conceptualization, Methodology, Supervision, Writing – original draft, Investigation, Writing – review & editing, Visualization, Formal analysis.

## Declaration of Competing Interest

The authors declare that they have no known competing financial interests or personal relationships that could have appeared to influence the work reported in this paper.
